# Growth kinetics of MPS-capped CdS quantum dots in self-assembled thin films

**DOI:** 10.1186/1556-276X-7-610

**Published:** 2012-11-05

**Authors:** Kenan Koç, Fatma Z Tepehan, Galip G Tepehan

**Affiliations:** 1 Department of Physics, Yildiz Technical University, Esenler, Istanbul, 34220, Turkey; 2 Department of Physics, Istanbul Technical University, Maslak, Istanbul, 34469, Turkey; 3 Faculty of Arts and Sciences, Kadir Has University, Fatih, Istanbul, 34083, Turkey

**Keywords:** CdS quantum dots, 3-Mercaptopropyltrimethoxysilane, Growth kinetics, Ostwald ripening, Thin films, 81.07.Ta, 81.16.Dn, 65.80.-g

## Abstract

For this study, we prepared colloidal CdS quantum dots using 3-mercaptopropyltrimethoxysilane as capping agent. Colloidal CdS quantum dots were directly deposited on glass substrates by a spin-coating process. Coated substrates were heat-treated between 225°C and 325°C for various heat treatment time intervals to investigate the growth kinetics of the quantum dots. Results showed that sizes of the CdS quantum dots grew approximately from 2.9 to 4.6 nm, and the *E*_1*s*1*s*_ energy values shifted approximately from 3.3 to 2.7 eV. Results showed that the average size of quantum dots increase by thermal treatment due to Ostwald ripening. The thermal process used to grow the size of quantum dots was examined according to the Lifshitz-Slyozov-Wagner theory. The activation energy of CdS quantum dots in thin films was calculated at approximately 44 kJ/mol.

## Background

Unlike bulk materials, optical and electronic properties of nanoparticles depend on their crystal dimension due to quantum confinement effect. Therefore, many research have focused on the production of nanomaterials that have controllable size [1-11]. Several techniques have been used to control the growth process of nanoparticles. One of the most important technique is the growth of nanoparticles in glasses. Generally, high heat treatment temperatures over 550°C were needed to control the growth process of nanoparticles for this technique [1-3]. Nanoparticles can also grow by chemical reactions in liquid or thin film media. Low heat treatment temperature and very narrow size distributions of nanoparticles in liquid or in thin film media are the obvious advantages compared with the growth of nanoparticles in glasses [4-11].

CdS thin films and nanoparticles have been widely used in the area of optoelectronics. CdS nanoparticles and thin films can be produced by several methods [10-13]. One of these is the use of capping agents to produce colloidal CdS quantum dots. 3-Mercaptopropyltrimethoxysilane (MPS) contains a bifunctional group molecule; the silane on one side can bond to the glass surface or metal oxide films, and the thiol group on the other side can work as a capping agent [11,12,14]. Addition of MPS in the solvent will serve as a precursor for the growth of SiO_2_ to incorporate quantum dots in a silica matrix [15]. SiO_2_ is an ideal shell material for CdS nanoparticles because SiO_2_ has a wide bandgap (9.1 eV) and an amorphous film structure [16].

Results show that the average size of quantum dots increases with the increasing heat treatment temperature and that small nanocrystals are dissolved with the increasing heat treatment temperature, and they are redeposited on the larger crystals due to Ostwald ripening. However, the effect of the capping agent thereby makes the growth process complex [17]. The amount of capping agent will affect the interfacial diffusion and hence the solubility of the nanocrystals. This phenomenon is attributed to the results on capped CdS quantum dots which were previously reported by Zhang et al. [18]. They said that ‘the growth of these QDs [quantum dots] was found to follow Ostwald ripening during the reflux process. When the amount of capping agent used exceeds some saturation value, an unprecedented dimerization of QDs is detected’. Fortunately, other effects can be neglected for Ostwald ripening if the appropriate amount of capping agent is used. Therefore, the amount of capping agent is selected in such a way that the bonding should neither be too strong nor too weak to allow for dynamic coupling in which the coordinating molecules attach and de-attach from growing nanoparticles [15].

In the present work, colloidal CdS quantum dots were directly deposited on glass substrates by spin-coating process. Therefore, self-assembled films made of CdS quantum dots in a SiO_2_ network were obtained using only one production step. The coated substrates were heat-treated between 225°C and 325°C for various time intervals to investigate the growth kinetics.

## Methods

### Production of quantum dots

CdS quantum dots were produced using the reaction of cadmium acetate (Cd(CH_3_COO)_2_·2H_2_O) with thioacetamide (CH_3_CSN_2_) at a molar ratio of one. Cadmium acetate and thioacetamide were first dissolved with methanol in separate beakers. The molar ratios of cadmium acetate/methanol and thioacetamide/methanol were equal to 0.02. MPS (HS(CH_2_)_3_Si(OCH_3_)_3_) was mixed with cadmium acetate at a molar ratio of MPS/Cd = 0.3 [12]. This ratio was decided after analyzing the absorption spectrum of the nano CdS at several other ratios (0.1, 0.2, 0.5) and after considering previous works [11,15,19]. The solutions in two beakers were then mixed and stirred for 10 min at 60°C in nitrogen medium.

### Production of thin films

MPS-capped CdS quantum dots were coated as thin film on a glass substrate (Corning 2947, Corning Incorporated, Tewksbury, MA, USA) by spin coating. The system was spun at a rate of 2,000 rev/min for 10 seconds. The substrate was heat-treated at 60°C for 10 min at each coating. The coated substrates were then heat-treated between 225°C and 325°C for various time intervals to investigate the growth kinetics. As explained in our previous work [12], during the formation of thin films, we suppose that quantum dots were attached to the glass surface by Si-O-Si bonds created by the OH group of the glass surface and the Si-O group of MPS.

### Characterization

High-resolution transmission electron microscopy (HRTEM) images and energy-dispersive X-ray spectroscopy (EDS) spectra of the MPS-capped CdS quantum dots in powder form were recorded using a JEOL 2100 (JEOL Ltd., Akishima, Tokyo, Japan) HRTEM microscope operated at 200 kV. X-ray photoelectron spectroscopy (XPS) spectra measurements of the thin films were carried out by SPECS PHOIBOS-150 Electron Analyzer (SPECS GmbH, Berlin, Germany) with a monochromatic X-ray source (Al-K*α*) of photon energy 1,486.6 eV. Absorbance of the thin films was recorded with a UV-visible spectrometer (Agilent 8453, Agilent Technologies, Inc., Santa Clara, CA, USA).

## Results and discussion

### HRTEM and EDS

**Figure 1:**
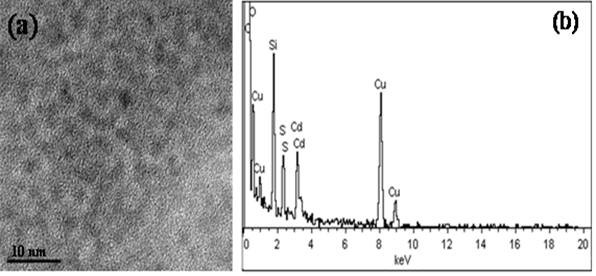
HRTEM image (a) and EDS pattern (b) of MPS-capped CdS quantum dots in powder form.

The HRTEM picture and the EDS pattern of MPS-capped CdS quantum dots are given in Figure 1a,b, respectively. In the EDS pattern, the presence of Cd and S is clear, and the atomic ratio of S/Cd was calculated as 1.07:1. In our work, cadmium acetate and thioacetamide were used as a Cd source and as an S source, respectively, in a molar ratio of 1 as explained in the ‘Methods’ section. On the other hand, MPS molecules contain sulfur due to their thiol group. Therefore, we think that S ratio is a little larger than that of Cd due to the contribution of the thiol group. Also, the oxygen and silicon peaks shown in the EDS pattern correspond to the capping agent of MPS.

### XPS spectra

**Figure 2:**
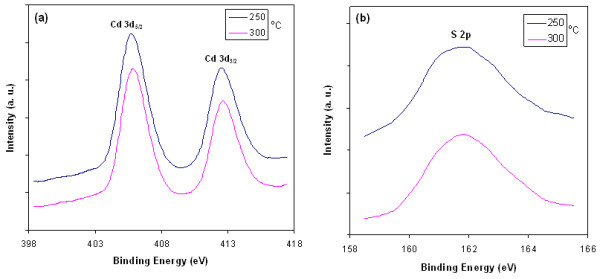
**XPS spectra of films heat-treated at 250°C and 300°C for 15 min.** (**a**) Cd 3d and (**b**) S 2p core level.

XPS spectra of the films heat-treated at 250°C and 300°C for 15 min are shown in Figure 2. In Figure 2a, it can be observed that Cd 3d_5/2_ peak at 405.73 eV and Cd 3d_3/2_ peak at 412.53 eV for the film heat-treated at 250°C for 15 min, while the binding energy peaks of Cd 3d_5/2_ and Cd 3d_3/2_ are observed at 405.88 and 412.63 eV for the film heat-treated at 300°C for 15 min. In Figure 2b, the S 2p peaks appear at 161.85 and 161.80 eV for the films heat-treated at 250°C and 300°C, respectively, for 15 min. All of the observed binding energy values are in good agreement with the reported data [20] and confirm the chemical identity of the CdS quantum dots. 

### Absorbance values

**Figure 3:**
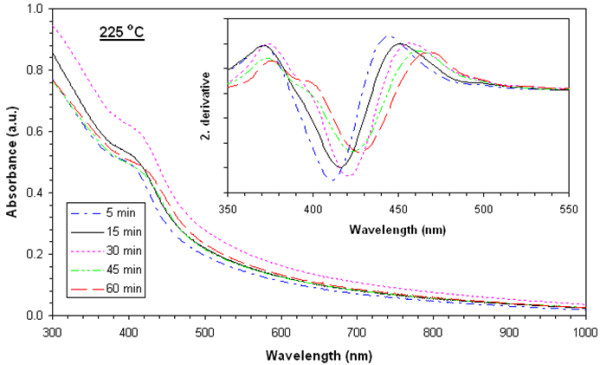
UV-visible absorption spectra of films heat-treated at 225°C at various times and corresponding second derivatives.

**Figure 4:**
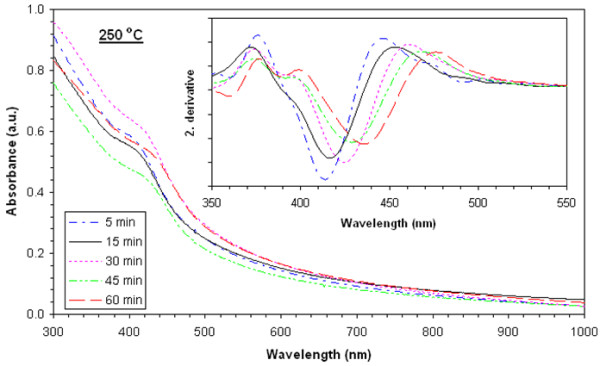
UV-visible absorption spectra of films heat-treated at 250°C at various times and corresponding second derivatives.

**Figure 5:**
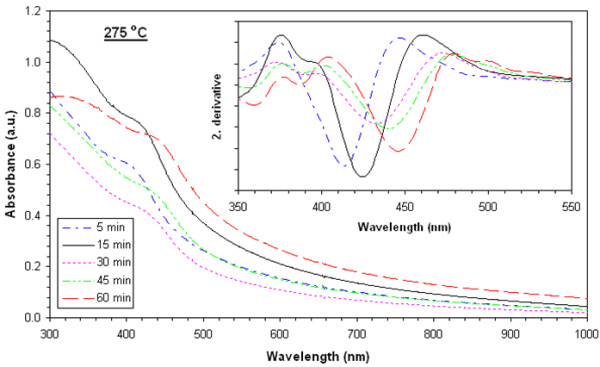
UV-visible absorption spectra of films heat-treated at 275°C at various times and corresponding second derivatives.

**Figure 6:**
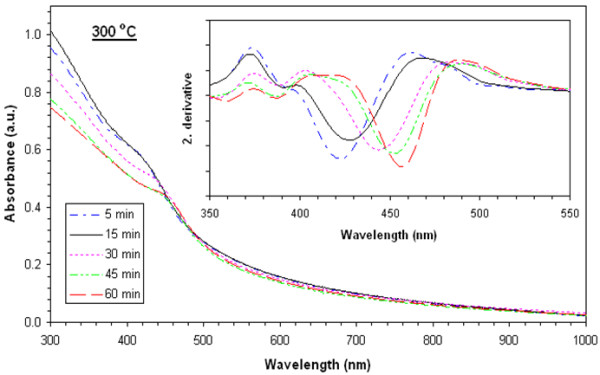
UV-visible absorption spectra of films heat-treated at 300°C at various times and corresponding second derivatives.

**Figure 7:**
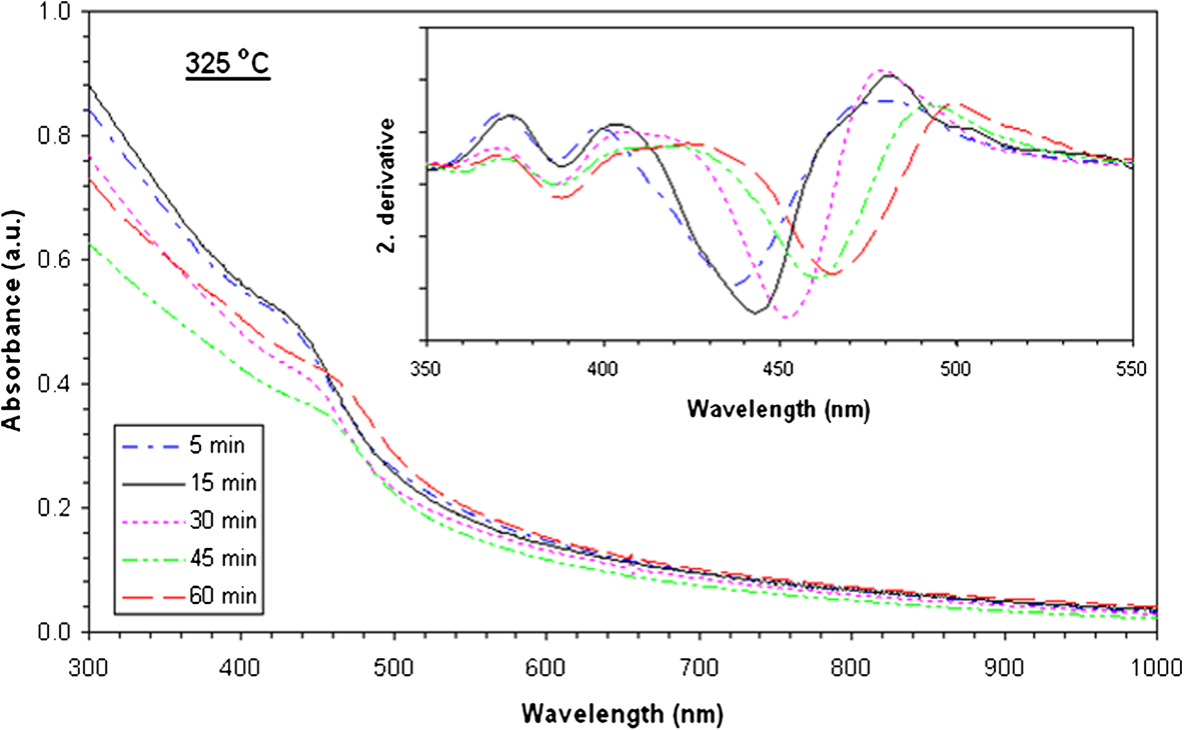
UV-visible absorption spectra of films heat-treated at 325°C at various times and corresponding second derivatives.

**Figure 8:**
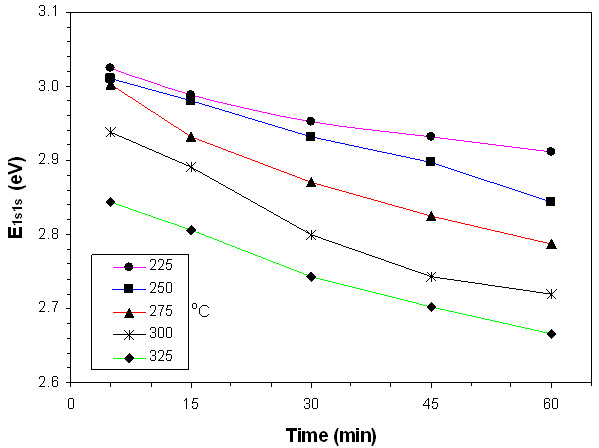
**The first exciton peak positions (*E***_**1*s*1*s***_**) against heat treatment time of films.**

Absorbance values of films heat-treated at 225°**C**, 250°**C**, 275°**C**, 300°**C**, and 325°C are shown in Figures 3, 4, 5, 6, and 7, respectively. The second derivatives of the absorbance graphs are also shown in the inset of the graphs. The figures show that absorbance edges are shifted through a larger wavelength (smaller energy) with respect to higher heat treatment time or temperature due to quantum confinement effects. The first exciton peak positions (*E*_1*s*1*s*_) are given in Figure 8; they were calculated from the first minimum value of the second derivatives of the absorbance graphs. *E*_1*s*1*s*_ energy values change between 3.02 and 2.67 eV, and as the time and temperature of the heat treatment increased, it approached to 2.42 eV being the bandgap energy of bulk CdS. In addition, as shown in the graphs, it is seen that as time of heat treatment increased, the shifting in *E*_1*s*1*s*_ values also increased. Shifting of *E*_1*s*1*s*_ values of films heat-treated at 225°C and 325°C for 5 min was 180 meV, and then it became 244 meV when the films were heat-treated at 225°**C** and 325°C for 60 min.

With the particle in a spherical box model, the first exciton peak position is given as follows [21,22]:

(1) $$E_{1s1s}=E_{g}+\frac{\hbar^{2}\pi^{2}}{2r^{2}}\left(\frac{1}{m_{e}^{*}}+\frac{1}{m_{h}^{*}}\right)-\frac{1.786e^{2}}{\varepsilon r}-0.248\frac{e^{4}}{2\varepsilon^{2}\hbar^{2}}{\left(\frac{1}{m_{e}^{*}}+\frac{1}{m_{h}^{*}}\right)}^{-1}\text{,}$$

where *E*_*g*_ is the bandgap energy of the bulk material (2.42 eV for CdS), *r* is the radius of the nanoparticle, *m*_e_^*^ and *m*_h_^*^ are the effective mass values of electrons and holes, respectively, (*m*_e_^*^ = 0.19*m*_0_ and *m*_h_^*^ = 0.8*m*_0_ for CdS), and ε is the dielectric constant (5.7 for CdS) [21]. The average particle size of these films is calculated by inserting *E*_1*s*1*s*_ values into Equation 1. The results of the calculations showed that the average *E*_1*s*1*s*_ values decreased from 3.3 eV (for films dried at 60°C for 10 min) to 2.7 eV (for films heat-treated at 325°C for 60 min), and sizes of the CdS quantum dots increased from 2.9 to 4.6 nm. The results show that the redshift of *E*_1*s*1*s*_ values with increasing heat treatment temperature is due to the increasing nanocrystal size (quantum size effect).

### Growth kinetics

The growth of nanocrystals can be described in terms of three stages: nucleation, normal growth stage, and Ostwald ripening. Size of nanocrystals is determined by the monomer concentration, capping ligand, and temperature at the nucleation and growth stages. At a fixed monomer concentration, Ostwald ripening dominates between nanoparticles. At this stage, the coarsening effects are characterized by diffusive mass transfer from smaller particles to larger ones, and the final size of nanocrystals is thus determined by temperature and time period of the Ostwald ripening [6]. We are going to look at the growth process of the films at this stage.

This diffusion-limited Ostwald ripening process can be described by the Lifshitz-Slyozov-Wagner (LSW) theory. The LSW theory for coarsening kinetics is given as follows [17]:

(2) $${\bar{r}}^{3}-{\bar{r}}_{0}^{3}=Kt\text{,}$$

where *r̄*_0_ is the average initial radius of nanocrystals, *r̄* is the average radius of nanocrystals after ripening occurs, *t* is the ripening time, and *K* is the ripening rate coefficient. *K* is given as follows [17]:

(3) $$K=\frac{8\gamma DV_{m}^{2}C_{\infty }}{9RT}\text{,}$$

where *D* is the diffusion coefficient, γ is the interfacial energy, *V*_*m*_ is the molar volume, *C*_∞_ is the equilibrium concentration at a flat surface, ℜ is the gas constant, and *T* is the temperature.

**Figure 9:**
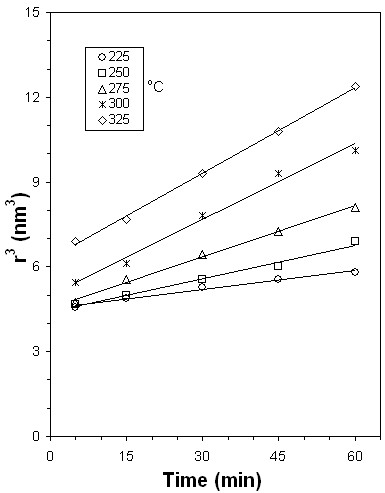
**The cubic term of*r̄*against heat treatment time of the films.**

The cubic term of *r̄* versus time of heat treatment are given in Figure 9. In this figure, for each temperature value, the linearly fitted curves are also given. It is seen that the results conform with the LSW theory given by Equation 2. For each heating temperature, *K* values were obtained from the slope of the linear curves. *K* values were calculated as 0.023, 0.039, 0.060, 0.090, and 0.106 nm^3^/min for the films heat-treated at 225°C, 250°C, 275°C, 300°C, and 325°C, respectively. As expected, these results show that for the Ostwald ripening process, the *K* values increased as the temperature increased. This shows that while the heat treatment temperature increased, the average radius of the quantum dots grew faster.

The relation between diffusion coefficient, activation energy, and temperature at constant pressure is given through the Arrhenius relation:

(4) $$D=D_{0}e^{-\frac{Q_{d}}{RT}}\text{,}$$

where *D*_0_ is a coefficient independent of temperature, ℜ is the gas constant, and *T* is the heat treatment temperature. *Q*_*d*_ is the (activation) energy of 1 mol of atom to make a diffusion movement.

**Figure 10:**
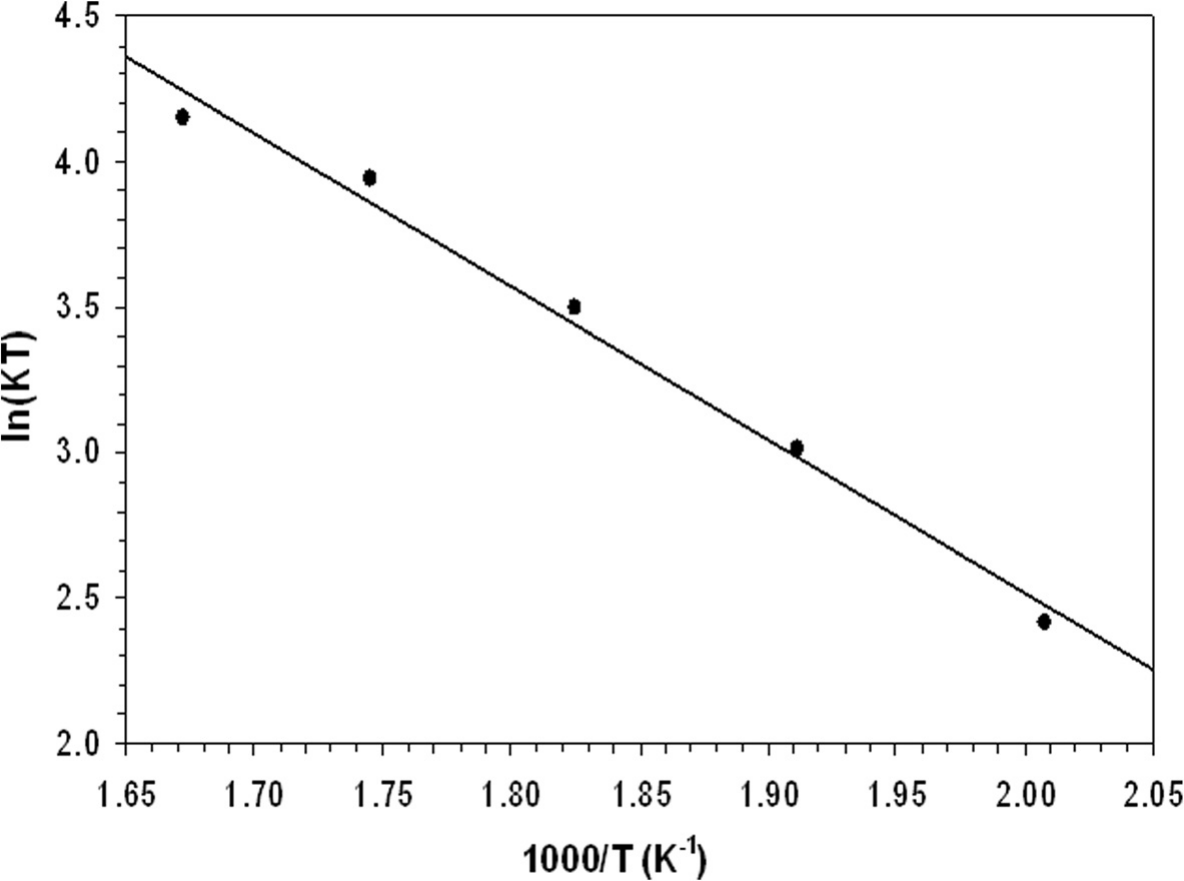
**The expression ln(*KT*) against 1,000/*T.***

Using Equations 3 and 4, the following equation for the activation energy is obtained:

(5) $$\ln\left(KT\right)=\ln\left(K_{0}\right)-\left(\frac{Q_{d}}{RT}\right)\text{,}$$

where *K*_0_ is a coefficient. In Figure 10, to find the activation energy from the slope of the fitted line, experimental points are ploted on the axis of ln(*KT*) with respect to 1,000/*T*. The activation energy for the Ostwald ripening process is found to be approximately 44 kJ/mol.

## Conclusions

In the present study, colloidal MPS-capped CdS quantum dots were self-assembled directly on a glass substrate using spin-coating method without introducing any matrix. The coated substrates were heat-treated to investigate the growth kinetics. Results showed that the average size of quantum dots changed from 2.9 to 4.6 nm when the heat treatment temperature and its duration time were increased. Compared to the growth of nanoparticles in glasses [2,3], the present growing method has the advantage of lower activation energy and lower heat treatment temperature, but the presence of a capping agent may prevent the growth to larger values. The applied techniques work well for CdS nanocrystalline particles and enable one to prepare a series of samples with well-defined particle radii.

## Competing interests

The authors declare that they have no competing interests.

## Authors’ contributions

KK carried out the preparation and characterization of the samples. KK, FZT, and GGT took part in the discussions of the results and prepared the manuscript. All authors read and approved the final manuscript.
